# The Science and Art of Obstetrics

**Published:** 1887-03

**Authors:** 


### Book Reviews.

The Science and Art of Obstetrics. *By* Theophilus Parvin, M.D., LL.D., *Professor of Obstetrics and Diseases of Women and Children in Jefferson Medical College, Philadelphia, etc., pp. xv. and 701. Philadelphia :* Lea Brothers & Company. *Chicago :* A. C. McClurg & Company. *1886.*

The author says in his preface that "he has endeavored to write a book which will be useful alike to students and to practitioners." He has been actively engaged as a teacher nearly twenty years, and may consequently be presumed to have included, within the limits which he has chosen, whatever he considers of essential value in relation to his subject. In its present edition, the book will hardly become a classic. As a practical guide for daily use by the under-graduates or by practitioners, it is excellent and reliable ; but as a book of reference, it will be found disappointing. All topics usually discussed in such books are very intelligently and practically treated in this one, and if disappointment arises in consulting it, it is because the treatment of the subject in hand is not quite full and complete. The book is fairly open to the charge that American authorities and experience are too exclusively drawn upon and cited.

The author's style is that of a teacher, rather than that of an author, and his book is much more practical than scientific. There are two hundred and fourteen wood-cuts and one colored plate, and these illustrations are fairly well chosen. The index, so far as tested, is satisfactory, and as already stated, the book will be found an excellent practical guide in the practice of obstetrics. E. W.

